# The assessment of correlation and prognosis among ^18^F-FDG uptake parameters, Glut1, pStat1 and pStat3 in surgically resected non-small cell lung cancer patients

**DOI:** 10.18632/oncotarget.25865

**Published:** 2018-08-10

**Authors:** Hayato Kaida, Koichi Azuma, Akihiko Kawahara, Eiji Sadashima, Satoshi Hattori, Shinzo Takamori, Jun Akiba, Kiminori Fujimoto, Axel Rominger, Takamichi Murakami, Kazunari Ishii, Masatoshi Ishibashi

**Affiliations:** ^1^ Department of Radiology, Kindai University Faculty of Medicine, Osakasayama, Osaka, Japan; ^2^ Division of Respirology, Department of Internal Medicine, Kurume University School of Medicine, Kurume, Fukuoka, Japan; ^3^ Department of Diagnostic Pathology, Kurume University Hospital, Kurume, Fukuoka, Japan; ^4^ Life Science, Saga-Ken Medical Centre Koseikan, Saga, Saga, Japan; ^5^ Department of Biomedical Statistics, Osaka University Graduate School of Medicine, Suita, Osaka, Japan; ^6^ Department of Surgery, Kurume University School of Medicine, Kurume, Fukuoka, Japan; ^7^ Department of Radiology, Kurume University School of Medicine, Kurume, Fukuoka, Japan; ^8^ Department of Nuclear Medicine, Inselspital, Bern University Hospital, Bern, Switzerland; ^9^ Department of Radiology, Kobe University Graduate School of Medicine, Kobe, Hyogo, Japan; ^10^ Department of Radiology, Fukuoka Tokushukai Medical Center, Kasuga, Fukuoka, Japan

**Keywords:** non-small cell lung cancer, volumetric parameters, Glut1, pStat1, pStat3

## Abstract

**Introduction:**

To assess the correlation among ^18^F-FDG uptake, Glut1, pStat1 and pStat3, and to investigate the relationship between the prognosis and ^18^F-FDG uptake and these molecular markers in surgically resected non-small cell lung cancer (NSCLC) patients.

**Results:**

Knockdown of Glut1 led to a significant increase in pStat1 expression. Glut1 expression positively correlated with the SUVmax, SUVmean, and TLG significantly (*P*<0.001). pStat3 expression negatively correlated with all PET parameters significantly (*P*<0.001). pStat1 had positive weak correlations with the SUVmax and SUVmean. All PET parameters and Glut1 were significantly associated with DFS (*P*<0.05). TLG, MTV, Glut1 and pStat1 were significantly associated with OS (*P*<0.05).

**Conclusion:**

pStat3 and Glut1 may be associated with ^18^F-FDG uptake mechanism. TLG, MTV, and Glut1 may be independent prognostic factors.

**Methods:**

The SUVmax, SUVmean, MTV and TLG of primary lesions were calculated in 140 patients. The expressions of Glut1 and Stat pathway proteins in NSCLC cell lines were examined by immune blots. Excised tumor tissue was analyzed by immunohistochemistry. OS and DFS were evaluated by the Kaplan-Meier method. The difference in survival between subgroups was analyzed by log-rank test. The prognostic significance of clinicopathological, molecular and PET parameters was assessed by Cox proportional hazard regression analysis.

## INTRODUCTION

Lung cancer is the leading cause of cancer death among men and women worldwide; an estimated 1.8 million new lung cancer cases occurred in 2012, accounting for approximately 13% of total cancer diagnoses [1]. The TNM staging system has been used as the most important prognostic factor for many types of cancer, but this staging system is considered not to predict the prognosis of non-small cell lung cancer (NSCLC) accurately, because it does not include molecular biological information.

The SUVmax has been reported to be an independent prognostic factor in NSCLC patients [2]. However, SUVmax reflects only the maximum glucose metabolism as measured in the highest pixels within a designated region of interest, which does not always reveal the glucose metabolism within the whole tumor. The TLG and the MTV are also taken into consideration as representative volumetric parameters to estimate the total radioactivity throughout a tumor above a minimum threshold, and these two parameters have been useful in predicting the prognosis and tumor response for malignant tumors [3, 4].

Glut1 expression contributes to the ^18^F-FDG uptake mechanism of malignant tumors because high glycolysis rate is observed in cancer cells. Stat1 is transferred to pStat1 by JAK phosphorylation, and the overexpression of the Stat1/INF pathway has been recently reported to be related with the resistance of chemotherapy and radiotherapy, metastasis, and poor prognosis in malignant tumors [5, 6].

Stat3 is activated by mutant EGFR and JAK, and notably, pStat3 is associated with tumor cell proliferation and angiogenesis [7]. To our knowledge, there are no reports regarding the correlation between ^18^F-FDG uptake and the Stat pathway, or investigations of the prognosis of NSCLC patients using volumetric parameters, Glut1, pStat1 and pStat3. We conducted the present study to assess the correlations between ^18^F-FDG uptake and Glut1, pStat1 and pStat3 and to investigate the relationship among the prognosis, volumetric parameters and molecular markers in completely resected NSCLC patients.

## RESULTS

### The correlation between PET parameters and clinicopathological variables

Our analyses revealed that TLG had significant positive correlations with the SUVmax (*R* = 0.723, *P* < 0.001), SUVmean (*R* = 0.697, *P* < 0.001) and MTV (*R* = 0.881, *P* < 0.001). The MTV also showed significant positive correlations with the SUVmax (*R* = 0.349, *P* < 0.001) and SUVmean (*R* = 0.306, *P* = 0.002). The details of the relationships between PET parameters and clinicopathological variables are summarized in Table 1. Patient age, gender, and pStage were each significantly associated with all PET parameters. Adjuvant chemotherapy was not associated with the SUVmean or SUVmax, but it was significantly associated with MTV (*P* = 0.011) and TLG (*P* = 0.008). Histology and smoking status were significantly associated with the SUVmax, SUVmean and TLG, but not with MTV. Only TLG showed significant associations with all of the clinicopathological variables.

**Table 1:** The relationship between ^18^F-FDG uptake and clinicopathological variables

Clinicopathological variables	SUVmax	P	SUVmean	P	MTV	P	TLG	P
Histology								
Adeno (*n*=114)	3.29(1.83,5.07)	<0.001	2.02(1.21,3.48)	<0.001	3.90(2.11,9.00)	<0.001	8.17(3.40,21.28)	0.002
Sq (*n*=26)	6.30(4.99,9.01)		3.94(3.02,5.54)		5.71(3.27,16.54)		27.57(8.12,77.94)	
Smoking status								
None (*n*=63)	2.67(1.68,4.89)	0.002	1.56(1.19,3.12)	0.002	3.42(2.08,8.35)	0.064	6.13(3.20,14.83)	0.004
Smoker (*n*=77)	4.76(2.53,6.62)		2.89(1.57,4.23)		4.75(2.62,14.12)		12.75(4.62,37.36)	
pStage								
Stage I (*n*=91)	2.68(1.81,5.67)	0.003	1.66(1.19,3.61)	0.003	3.39(2.08,7.73)	0.003	6.13(3.45,17.41)	<0.001
Stage II,III (*n*=49)	4.88(3.40,6.47)		3.02(2.07,4.22)		6.75(3.20,15.96)		20.38(8.12,51.51)	
Adjuvant therapy								
Absent (*n*=88)	3.11(1.85,5.97)	0.157	1.91(1.19,3.80)	0.219	3.37(2.21,6.98)	0.011	6.36(3.94,20.38)	0.008
Present(*n*=52)	4.41(2.67,6.18)		2.81(1.54,3.98)		8.29(2.69,14.12)		19.31(5.24,37.36)	
Gender								
Men (*n*=85)	4.76(2.32,6.62)	0.001	2.89(1.52,4.23)	0.002	4.93(2.59,15.26)	0.03	12.75(4.20,50.55)	0.003
Women (*n*=55)	2.67(1.68,4.82)		1.59(1.19,3.03)		3.32(2.08,8.35)		5.86(3.49,14.14)	
Age								
<73 (*n*=66)	3.07(1.83,4.99)	0.002	1.90(1.20,3.21)	0.005	3.28(2.10,7.43)	0.011	5.72(3.10,16.94)	<0.001
73≥– (*n*=74)	4.82(2.21,7.34)		2.96(1.40,4.38)		4.83(2.98,14.69)		14.14(5.10,34.24)	

### Effects of Glut1 knockdown on the expression of Stat pathway proteins in human NSCLC Cells

Given the significant correlations between ^18^F-FDG uptake and Glut1 expression in this analysis and a previous study [8], we examined the expression of Glut1 in lung cell lines. A high expression of Glut1 was observed in the HCC827, EBC1, NCI-H1993, and Calu1cell lines, but not in the other cell lines.

**Figure 1:**
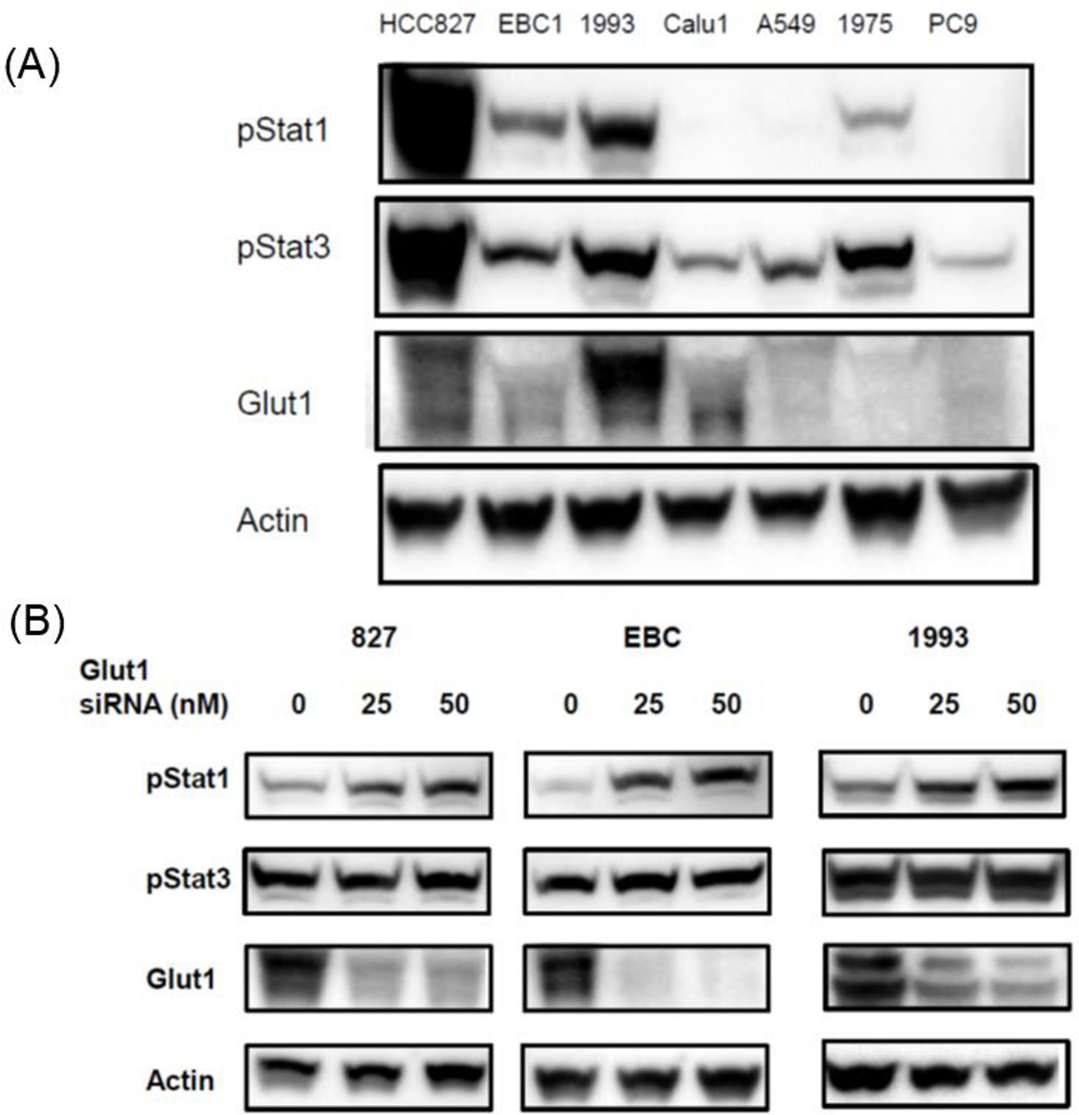
Effects of Glut1 knockdown on the expression of Stat signal pathway proteins in human lung cell lines **(A)** A high expression of Glut1 was observed in the HCC827, EBC1, NCI-H1993, and Calu1cell lines. **(B)** Transient knockdown by the transfection of HCC827, EBC1, and NCI-H1993 cells with Glut1 siRNA increased the expressions of pStat1, but did not change the expression of pStat3.

We then examined the effect of Glut1 knockdown using cognate siRNA on the expressions of pStat1 and pStat3 proteins (Figure 1A). Transient knockdown by the transfection of HCC827, EBC1, and NCI-H1993 cells with Glut1 siRNA increased the expressions of pStat1, but did not change the expression of pStat3 (Figure 1B). These results indicate that Glut1 is associated with the Stat signal pathway in human lung cancer cells.

### The correlations between PET parameters and molecular markers

**Figure 2:**
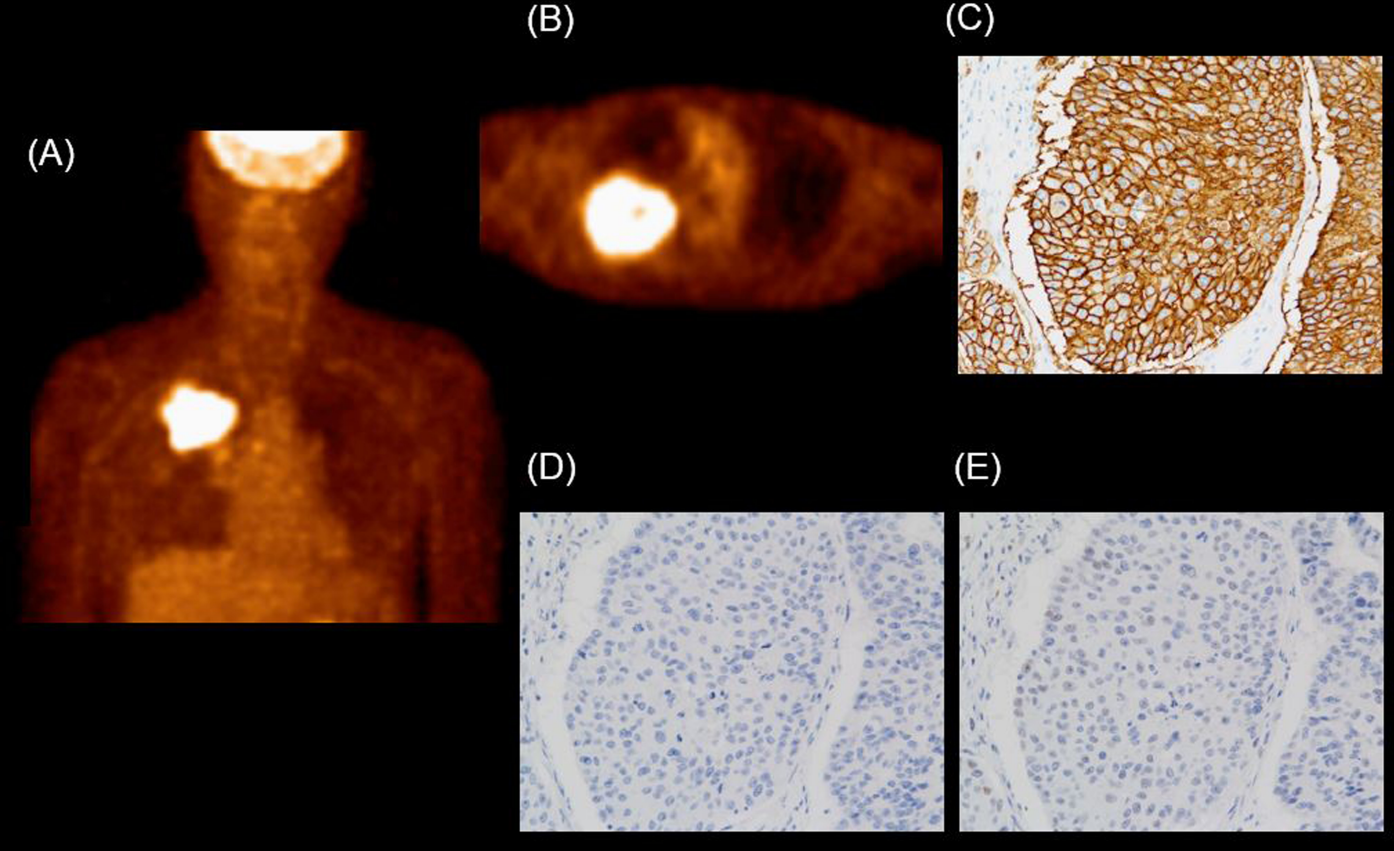
Example of NSCLC (adenocarcinoma; pT2N0M0 stage IIA) in an 81-yr-old man High ^18^F-FDG uptake is observed in the right upper lung field. **(A)** MIP image. **(B)** Axial PET image. SUVmean: 5.39, SUVmax: 8.30, MTV: 110.3, and TLG: 595.31. **(C)** The Glut1 score was 210. **(D)** The pStat1 expression score was 1. **(E)** The pStat3 expression score was 30.

The relationships between ^18^F-FDG parameters and Glut1, pStat1, and pStat3 are summarized in Table 2. The Glut1 expression had significant positive correlations with the SUVmax (*R* = 0.62, *P* < 0.001), SUVmean (*R* = 0.611, *P* < 0.001), and TLG (*R* = 0.359, *P* < 0.001), but not with MTV. The pStat3 expression had significant negative correlations with the SUVmax (*R* = -0.577, *P* < 0.001), SUVmean (*R*= -0.586, *P* < 0.001), MTV (*R*= -0.215, *P* < 0.001), and TLG (*R*= -0.435 *P* < 0.001). The pStat1 expression showed significant positive correlations with the SUVmax and SUVmean, but not with MTV and TLG. A representative patient's case is shown in Figure 2.

**Table 2:** The relationship between ^18^F-FDG uptake and molecular markers

Molecular biological markers	Glut1	P	pStat1	P	pStat3	P
SUVmax	R=0.62	<0.001	R=0.171	0.043	R=-0.577	<0.001
SUVmean	R=0.611	<0.001	R=0.168	0.046	R=-0.568	<0.001
MTV	R=0.103	0.224	R=0.051	0.546	R=-0.215	0.017
TLG	R=0.359	<0.001	R=0.116	0.171	R=-0.435	<0.001

### Associations among PET parameters, molecular markers, and survival

**Figure 3:**
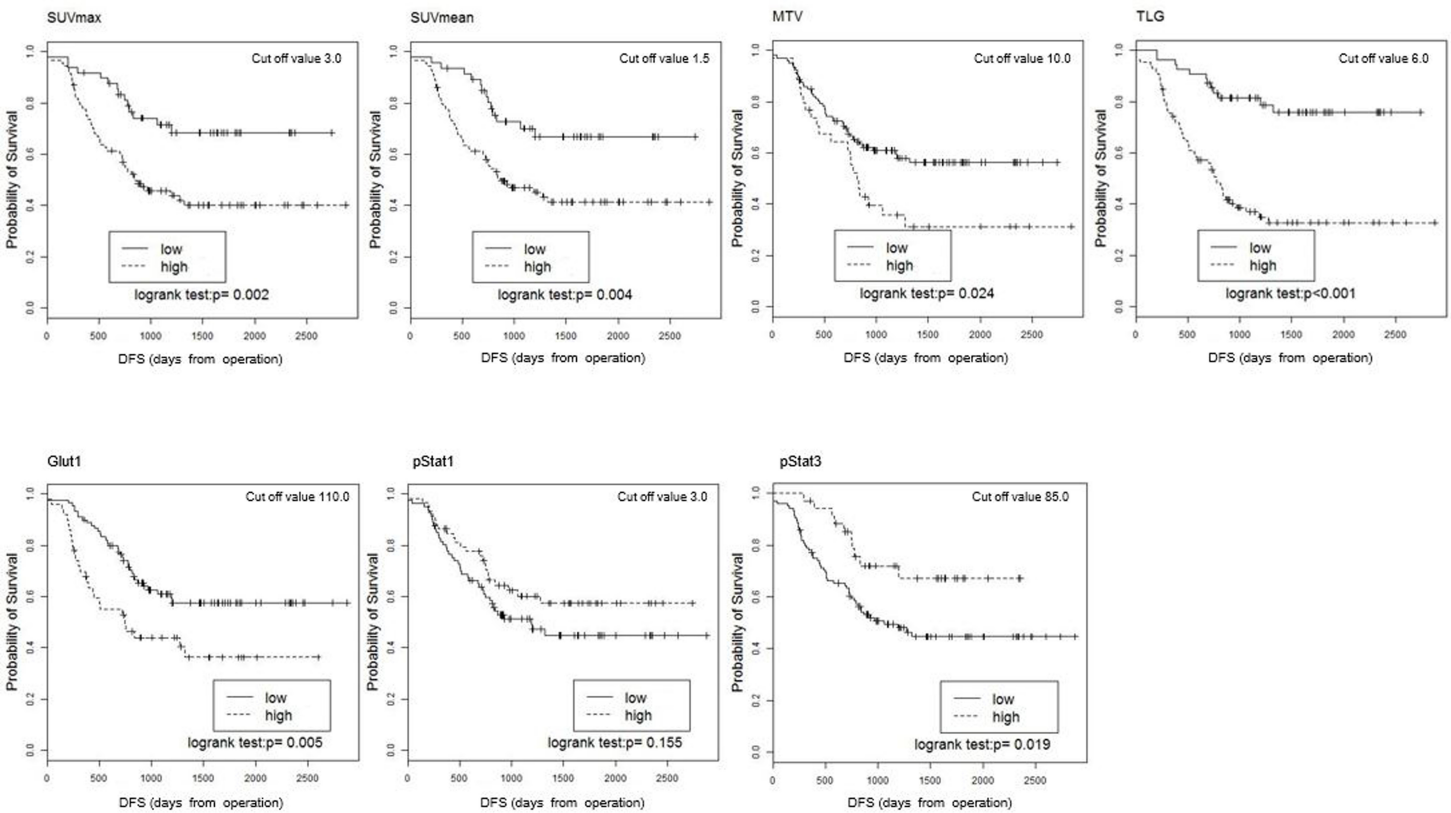
Kaplan-Meier estimates of survival functions for DFS in the low and high groups for the SUVmean, SUVmax, MTV, TLG, Glut1, pStat1, and pStat3 in the 140 NSCLC patients P-values were determined by the log-rank test.

**Figure 4:**
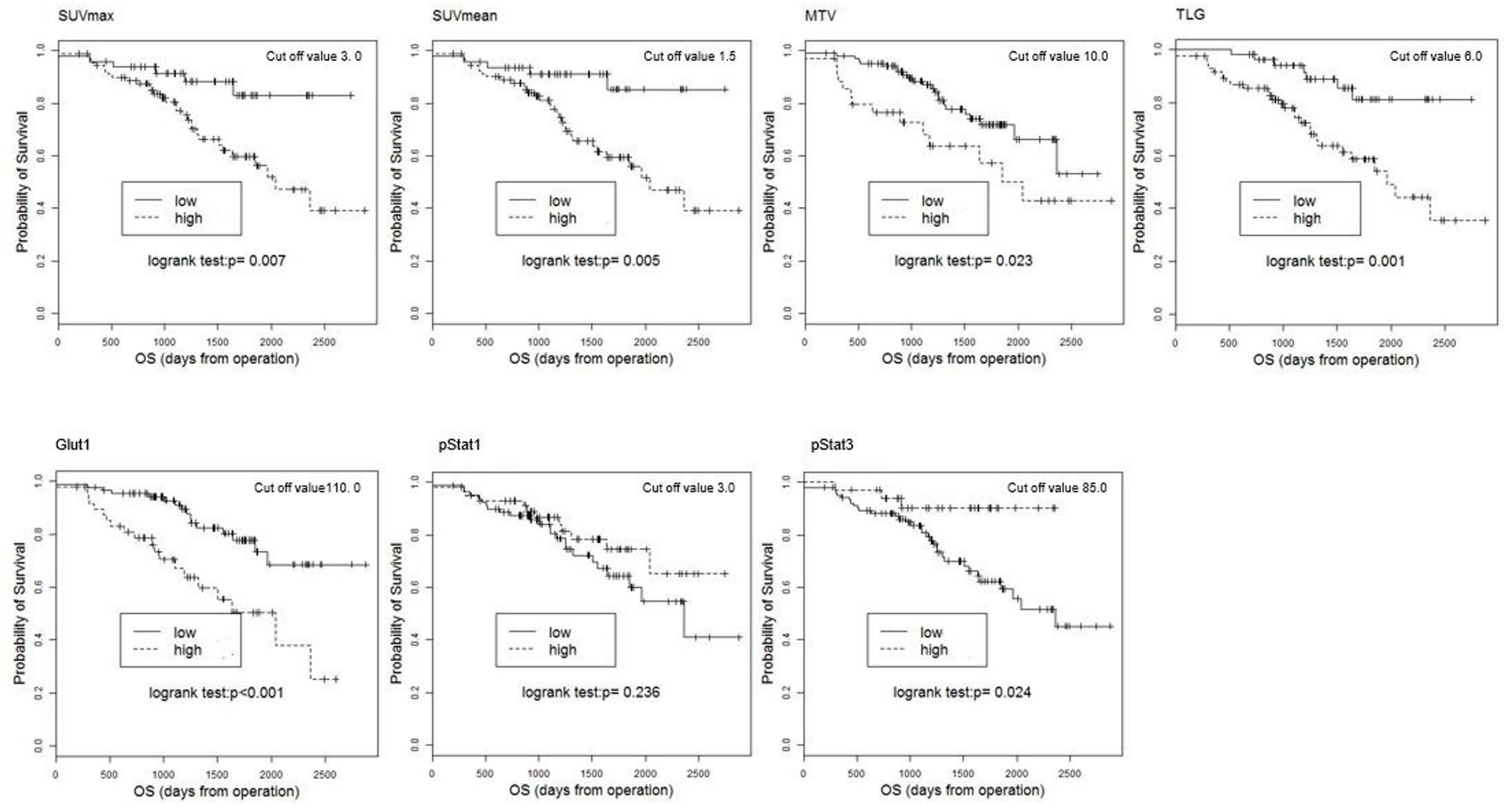
Kaplan-Meier estimates of survival functions for OS in the low and high groups for the SUV mean, SUVmax, MTV, TLG, Glut1, pStat1, and pStat3 in 140 NSCLC patients P-values were determined by the log-rank test.

The median follow-up period was 1,198 days (range 192–2,834 days). Sixty-five of the 140 patients (46.1%) developed local recurrence or distant metastases, and 37 (26.2%) of the 140 patients died during the study period. The Kaplan-Meier survival curves for DFS and OS are shown (Figures 3 and 4). The optimal cut-off values for what were as follows: SUVmax, 3.0; SUVmean, 1.5; MTV, 10.0; TLG, 6.0; Glut1 score, 110; pStat1 score, 3.0; and pStat3 score, 85. On both the DFS and OS for Kaplan-Meier survival curves, the survival curves of the groups of patients with low SUVmean, low SUVmax, low MTV, and low TLG are significantly longer than those of the high groups, as follows. SUVmean: DFS, *P* = 0.004, OS, *P =* 0.005; SUVmax: DFS, *P* = 0.002, OS, *P* = 0.007; MTV: DFS, *P* = 0.024, OS, *P* = 0.023; TLG: DFS, *P* < 0.001, OS, *P* = 0.001. The survival curves of the patients with high Glut1 or low pStat3 expression were significantly shorter than those of the patients with low Glut1 or high pStat3, with the following values. Glut1: DFS, *P* = 0.005, OS, *P* < 0.001; pStat3: DFS, *P* = 0.019, OS, *P* = 0.024. There was no significant difference in DFS or OS between the low-pStat1 expression group and the high-pStat1 expression group.

The details of the results of the Cox proportional hazard model for OS and DFS are provided (Tables 3 and 4). After adjusting for potential confounding factors, all PET parameters and Glut1 remained significantly correlated with DFS (SUVmax: HR = 1.443: 95% CI 1.023–2.034, *P* = 0.037; SUVmean: HR = 1.404, 95%CI 1.013–1.946, *P* = 0.042; MTV: HR = 1.199, 95%CI 1.085–1.324, *P* < 0.001; TLG: HR = 1.134, 95%CI 1.064–1.208, *P* < 0.001; Glut1: HR = 3.636, 95%CI 1.981–6.673, *P* < 0.001). Regarding OS, our analyses revealed that TLG, MTV, Glut1 and pStat1 remained significantly correlated with the OS (MTV: HR = 1.244, 95%CI 1.111–1.393, *P* < 0.001; TLG: HR = 1.172, 95%CI 1.083–1.268, *P* < 0.001; Glut1: HR = 3.569, 95%CI 1.534–8.304, *P* = 0.003; pStat1: HR = 1.260, 95%CI, 1.022–1.553; *P* = 0.031). pStat1 and pStat3 were not significantly related to the DFS (pStat1: HR = 1.099, 95%CI, 0.957–1.262; *P* = 0.18, pStat3: HR = 0.779, 95%CI, 0.511–1.188; *P* = 0.246), and the SUVmax, SUVmean, and pStat3 were not significantly associated with the OS (SUVmax: HR = 1.227, 95%CI, 0.738–2.039; *P* = 0.431, SUVmean: HR = 1.232, 95%CI, 0.753–2.016; *P* = 0.405, pStat3: HR = 0.815, 95%CI, 0.421–1.578; *P* = 0.544).

**Table 3:** Results of Cox regression analysis for DFS

	DFS (Full model)		DFS (Reduced model)	
	HR(95%CI)	P	HR(95%CI)	P
SUVmax(Unit=q3-q1)	1.377( 0.962, 1.970)	0.08	1.443( 1.023, 2.034)	0.037
pStage (II,III/I)	2.068( 1.213, 3.524)	0.008	2.170( 1.311, 3.590)	0.003
Adjuvant (Present/Absent)	1.411( 0.812, 2.455)	0.222		
Age (Unit=10)	1.307( 0.985, 1.736)	0.064	1.229( 0.940, 1.607)	0.131
Gender (Women/Men)	0.760( 0.386, 1.493)	0.425		
Smoking (Smoker/Never)	1.607( 0.864, 2.990)	0.134		
Histology (Adeno/Sq)	1.821( 0.864, 3.836)	0.115	1.558( 0.728, 3.336)	0.253
SUVmean(Unit=q3-q1)	1.331( 0.953, 1.861)	0.094	1.404( 1.013, 1.946)	0.042
pStage (II,III/I)	2.109( 1.241, 3.585)	0.006	2.222( 1.348, 3.662)	0.002
Adjuvant (Present/Absent)	1.409( 0.810, 2.450)	0.225		
Age (Unit=10)	1.311( 0.988, 1.740)	0.06	1.232( 0.943, 1.610)	0.126
Gender (Women/Men)	0.743( 0.379, 1.455)	0.386		
Smoking (Smoker/Never)	1.581( 0.848, 2.946)	0.149		
Histology (Adeno/Sq)	1.800( 0.855, 3.790)	0.122	1.537( 0.720, 3.279)	0.267
MTV(Unit=q3-q1)	1.156( 1.038, 1.287)	0.008	1.199( 1.085, 1.324)	<0.001
pStage (II,III/I)	1.990( 1.155, 3.430)	0.013	2.042( 1.221, 3.413)	0.006
Adjuvant (Present/Absent)	1.275( 0.726, 2.238)	0.398		
Age (Unit=10)	1.280( 0.963, 1.700)	0.089	1.228( 0.940, 1.604)	0.133
Gender (Women/Men)	0.747( 0.381, 1.467)	0.397		
Smoking (Smoker/Never)	1.460( 0.776, 2.745)	0.241		
Histology (Adeno/Sq)	1.370( 0.686, 2.735)	0.372	1.101( 0.567, 2.134)	0.777
TLG(Unit=q3-q1)	1.108( 1.036, 1.185)	0.003	1.134( 1.064, 1.208)	<0.001
pStage (II,III/I)	2.036( 1.182, 3.507)	0.01	2.088( 1.254, 3.475)	0.005
Adjuvant (Present/Absent)	1.273( 0.726, 2.234)	0.4		
Age (Unit=10)	1.276( 0.962, 1.693)	0.09	1.226( 0.938, 1.602)	0.135
Gender (Women/Men)	0.757( 0.386, 1.484)	0.418		
Smoking (Smoker/Never)	1.480( 0.788, 2.779)	0.223		
Histology (Adeno/Sq)	1.425( 0.714, 2.841)	0.315	1.156( 0.597, 2.238)	0.667
Glut1score(High/Low)	3.098( 1.622, 5.917)	0.001	3.636( 1.981, 6.673)	<0.001
pStage (II,III/I)	2.235( 1.295, 3.856)	0.004	2.484( 1.510, 4.088)	<0.001
Adjuvant (Present/Absent)	1.366( 0.777, 2.403)	0.279		
Age (Unit=10)	1.382( 1.039, 1.840)	0.026	1.309( 1.002, 1.711)	0.048
Gender (Women/Men)	0.787( 0.395, 1.568)	0.496		
Smoking (Smoker/Never)	1.319( 0.694, 2.508)	0.398		
Histology (Adeno/Sq)	3.730( 1.596, 8.722)	0.002	3.730( 1.599, 8.703)	0.002
pStat1score(High/Low)	1.124( 0.963, 1.312)	0.137	1.099( 0.957, 1.262)	0.18
pStage (II,III/I)	2.193( 1.297, 3.706)	0.003	2.377( 1.449, 3.901)	0.001
Adjuvant (Present/Absent)	1.405( 0.811, 2.436)	0.225		
Age (Unit=10)	1.308( 0.984, 1.737)	0.064	1.255( 0.961, 1.638)	0.096
Gender (Women/Men)	0.576( 0.289, 1.150)	0.118		
Smoking (Smoker/Never)	1.357( 0.714, 2.581)	0.351		
Histology (Adeno/Sq)	1.511( 0.753, 3.034)	0.246	1.108( 0.571, 2.148)	0.762
pStat3score(High/Low)	0.879( 0.567, 1.363)	0.565	0.779( 0.511, 1.188)	0.246
pStage (II,III/I)	2.152( 1.259, 3.677)	0.005	2.244( 1.359, 3.706)	0.002
Adjuvant (Present/Absent)	1.358( 0.779, 2.369)	0.281		
Age (Unit=10)	1.362( 1.031, 1.800)	0.03	1.301( 0.996, 1.699)	0.053
Gender (Women/Men)	0.706( 0.352, 1.419)	0.329		
Smoking (Smoker/Never)	1.575( 0.831, 2.984)	0.164		
Histology (Adeno/Sq)	1.505( 0.749, 3.026)	0.251	1.211( 0.615, 2.385)	0.58

**Table 4:** Results of Cox regression analysis for OS

	OS (Full model)		OS (Reduced model)	
	HR(95%CI)	P	HR(95%CI)	P
SUVmax(Unit=q3-q1)	1.232( 0.718, 2.113)	0.449	1.227( 0.738, 2.039)	0.431
pStage (II,III/I)	2.861( 1.330, 6.155)	0.007	2.383( 1.200, 4.729)	0.013
Adjuvant (Present/Absent)	0.781( 0.355, 1.717)	0.539		
Age (Unit=10)	1.068( 0.735, 1.553)	0.73		
Gender (Women/Men)	0.386( 0.134, 1.114)	0.078	0.283( 0.118, 0.681)	0.005
Smoking (Smoker/Never)	1.785( 0.716, 4.449)	0.214		
Histology (Adeno/Sq)	1.592( 0.645, 3.930)	0.313	1.396( 0.578, 3.369)	0.459
SUVmean(Unit=q3-q1)	1.224( 0.732, 2.047)	0.44	1.232( 0.753, 2.016)	0.405
pStage (II,III/I)	2.885( 1.348, 6.172)	0.006	2.396( 1.214, 4.729)	0.012
Adjuvant (Present/Absent)	0.777( 0.354, 1.708)	0.53		
Age (Unit=10)	1.068( 0.734, 1.552)	0.732		
Gender (Women/Men)	0.381( 0.133, 1.092)	0.072	0.283( 0.118, 0.678)	0.005
Smoking (Smoker/Never)	1.765( 0.710, 4.389)	0.221		
Histology (Adeno/Sq)	1.586( 0.646, 3.895)	0.314	1.397( 0.581, 3.360)	0.455
MTV(Unit=q3-q1)	1.255( 1.111, 1.416)	<0.001	1.244( 1.111, 1.393)	<0.001
pStage (II,III/I)	2.661( 1.216, 5.826)	0.014	2.076( 1.034, 4.167)	0.04
Adjuvant (Present/Absent)	0.645( 0.286, 1.455)	0.29		
Age (Unit=10)	0.959( 0.662, 1.390)	0.826		
Gender (Women/Men)	0.394( 0.141, 1.103)	0.076	0.325( 0.136, 0.773)	0.011
Smoking (Smoker/Never)	1.555( 0.622, 3.889)	0.345		
Histology (Adeno/Sq)	1.366( 0.588, 3.172)	0.468	1.236( 0.538, 2.840)	0.618
TLG(Unit=q3-q1)	1.177( 1.083, 1.280)	<0.001	1.172( 1.083, 1.268)	<0.001
pStage (II,III/I)	2.657( 1.209, 5.838)	0.015	2.047( 1.019, 4.113)	0.044
Adjuvant (Present/Absent)	0.647( 0.288, 1.457)	0.294		
Age (Unit=10)	0.966( 0.668, 1.397)	0.854		
Gender (Women/Men)	0.401( 0.145, 1.111)	0.079	0.331( 0.139, 0.790)	0.013
Smoking (Smoker/Never)	1.594( 0.642, 3.960)	0.315		
Histology (Adeno/Sq)	1.416( 0.614, 3.266)	0.415	1.249( 0.549, 2.842)	0.596
Glut1score(High/Low)	3.246( 1.389, 7.585)	0.007	3.569( 1.534, 8.304)	0.003
pStage (II,III/I)	2.744( 1.293, 5.821)	0.009	2.429( 1.249, 4.727)	0.009
Adjuvant (Present/Absent)	0.824( 0.373, 1.821)	0.632		
Age (Unit=10)	1.132( 0.781, 1.639)	0.513		
Gender (Women/Men)	0.437( 0.152, 1.257)	0.125	0.374( 0.152, 0.921)	0.032
Smoking (Smoker/Never)	1.376( 0.544, 3.480)	0.5		
Histology (Adeno/Sq)	3.236( 1.201, 8.720)	0.02	3.146( 1.169, 8.472)	0.023
pStat1score(High/Low)	1.222( 0.980, 1.524)	0.075	1.260( 1.022, 1.553)	0.031
pStage (II,III/I)	3.110( 1.485, 6.514)	0.003	2.725( 1.396, 5.322)	0.003
Adjuvant (Present/Absent)	0.755( 0.343, 1.663)	0.485		
Age (Unit=10)	1.039( 0.722, 1.495)	0.838		
Gender (Women/Men)	0.266( 0.090, 0.785)	0.017	0.226( 0.093, 0.551)	0.001
Smoking (Smoker/Never)	1.473( 0.603, 3.597)	0.396		
Histology (Adeno/Sq)	1.475( 0.635, 3.428)	0.366	1.328( 0.579, 3.048)	0.503
pStat3score(High/Low)	0.769( 0.393, 1.507)	0.444	0.815( 0.421, 1.578)	0.544
pStage (II,III/I)	2.959( 1.391, 6.292)	0.005	2.460( 1.257, 4.814)	0.009
Adjuvant (Present/Absent)	0.758( 0.345, 1.665)	0.49		
Age (Unit=10)	1.106( 0.773, 1.582)	0.582		
Gender (Women/Men)	0.395( 0.133, 1.171)	0.094	0.282( 0.117, 0.680)	0.005
Smoking (Smoker/Never)	1.786( 0.703, 4.537)	0.223		
Histology (Adeno/Sq)	1.414( 0.616, 3.250)	0.414	1.239( 0.544, 2.823)	0.609

## DISCUSSION

The amount of ^18^F-FDG accumulation has been reported to be associated with molecules relevant to glucose metabolism, hypoxia (HIF-1α), angiogenesis (CD43, VEGF) and mTOR signaling pathway, and PTEN in NSCLC patients [8]. The correlation between ^18^F-FDG uptake in primary tumor and these molecular markers has been examined in head neck cancer, pancreas cancer and uterine cervical cancer patients [9–11]. Mano et al. demonstrated that ^18^F-FDG uptake is associated with the expressions of pStat3, HIF-1α and Glut1 in hepatocellular carcinoma, and that pStat3 had a significant positive correlation with ^18^F-FDG accumulation [12]. However, to our knowledge, the relationship between ^18^F-FDG uptake mechanism and pStat pathway and the correlation between Glut1 and pStat pathway have not been clarified in NSCLC patients. We examined the effect of Glut1 knockdown using cognate siRNA on the expression of pStat proteins. The transient knockdown by the transfection of NSCLC cells with Glut1 siRNA increased the expression of pStat1. Based on those results, we conducted an immunohistochemical staining examination of Glut1, pStat1 and pStat3 in the 140 NSCLC patients.

The SUVmax, SUVmean and TLG each had a significant positive correlation with Glut1. All PET parameters had a significant inverse correlation with pStat3 expression. Koh et al. reported that high values of volumetric parameters were significantly higher in Glut1-positive compared to Glut1-negative adenocarcinoma [13]. Harura et al. suggested that high pStat3 expression was related to smaller tumor size, limited smoking, and an anti-apoptosis effect on early NSCLC, and that agents targeting the EGFR-Stat3 pathway may have better efficiency in early-stage NSCLC patients compared to advanced-stage patients [14]. The inverse correlation between ^18^F-FDG uptake and pStat3 expression may be due to the EGFR-Stat3 pathway. Unfortunately, these correlations between pStat1 and SUVmax and SUVmean are weak, and volumetric parameters did not correlate with pStat1. pStat3 and Glut1 expression rather than pStat1 expression may modulate the ^18^F-FDG uptake in NSCLC.

Research regarding a pStat3 inhibitor, OPB-51602, has been progressing [15]. ^18^F-FDG uptake may be a promising biomarker for predicting pStat3 expression in response to molecular target therapy. However, our data about correlation between^18^F-FDG accumulation and pStat3 expression was not agreement with Mano et al. [12]. The correlation between pStat3 expression and ^18^F-FDG uptake may differ by the type of cancer. This correlation should be investigated in other malignant tumors.

Volumetric parameters have been reported to be useful to predict the tumor response and the prognosis after chemotherapy in advanced NSCLC [16]. Regarding preoperative NSCLC patients, Kim et al. reported that the MTV was the only significant prognostic factor for OS in their multivariate analysis of stage I–IIIA patients [17]. Hyum et al. found that in addition to pathological TNM stage, volumetric parameters were independent prognostic factors for both OS and DFS in stage I–II patients [18]. TLG was a significant prognostic factor for OS in stage I patients, as described by Park et al. [19]. Our present findings are in agreement with Hyum et al. [18]. However, Domachevsky et al. reported that the SUVmax was a more useful independent prognostic marker than volumetric parameters in patients with stage I–II NSCLC, and Lin et al. suggested that the SUVmax was the only parameter for the DFS of patients with stage I NSCLC [20, 21]. The usefulness of volumetric parameters for predicting the prognosis of patients with resected NSCLC thus remains controversial and should be investigated in greater numbers of patients.

The advantage of this study compared to prior investigations is that we examined the prognosis of NSCLC using four PET parameters and molecular markers, and we revealed the correlations between ^18^F-FDG uptake and molecular markers using immunohistochemical staining examination and immune blots. Our present study demonstrated that Glut1 is a prognostic factor for NSCLC patients. Nguyen et al. reported that the ^18^F-FDG uptake is more valuable than Glut1 or Ki-67 expression for predicting the prognosis of resected NSCLC patients [22]. Kaira et al. stated that SUVmax and Glut1 were independent prognostic factors for DFS in adenocarcinoma patients [8]. We consider that TLG, MTV and Glut1 are significant prognostic factors for DFS and OS in resected NSCLC patients.

In the present patients, pStat3 was not shown to be an independent prognostic factor. Galleges Ruiz et al. suggested that (1) the EGFR-Stat3 pathway may be related to the tumor growth of early NSCLC (2) pStat3-positive expression in NSCLC may indicate the indolent type, and (3) high pStat3 expression was significantly associated with longer OS and PFS [20]. Our data are partially in agreement with those of Galleges Ruiz et al. [23]. However, Xu et al. noted that high pStat3 expression is a strong predictor of poor prognosis among NSCLC patients [24]. This issue remains to be clarified.

pStat1 has been reported to be associated with poor prognosis in malignant tumors, and with chemotherapy resistance in lung cancer [7, 25, 26]. In the present study, we observed a significant difference between pStat1 expression and OS in the multivariate analysis, but not between pStat1 expression and DFS. We speculate that pStat1 expression may be a significant prognostic factor for the OS of NSCLC patients, although pStat1 does not contribute to the ^18^F-FDG uptake mechanism.

There are some study limitations to consider. First, the study was retrospective, and our patient distribution was heterogeneous because of the inclusion of stage I–IIIA patients and those who had undergone postoperative adjuvant therapy. We performed the statistical analysis using adjusted clinicopathological prognostic variables. Second, the measurement of the MTV has not been standardized. There are several tumor delineation methods for the MTV, which includes a manual method, an automatic method and a semiautomatic method. Fixed-threshold methods such as the use of the fixed cut-off SUVmax of 2.5 and a fixed percentage of SUVmax thresholds (typically 40%–80%) have been used [27]. We used the threshold of 40% of SUVmax, because this method was used in the literature [28]. The fixed cut-off SUVmax of 2.5 for measuring ^18^F-FDG uptake lesions for MTV has been used in past investigations [17, 19]. However, ^18^F-FDG uptake lesions of NSCLC less than SUVmax 2.5 exist in clinical setting, and the chance of malignant lung nodule less than SUVmax 2.5 has been reported to be 62% [29]. In this study, we used the threshold 40% of SUVmax, because forty-eight of all 140 NSCLC lesions (34%) were less than SUVmax 2.5. In addition, Laffron et al. suggested that a low threshold should be suitable for predicting treatment effects and survival in lung cancer patients, because the TLG variability is greater at high thresholds [27]. Paidpally et al. recently reported that there is excellent inter-reader agreement for the measurement of the MTV and TLG with 40% and 50% SUVmax threshold segmentation [30]. The issue of which tumor delineation methods and cut-off values are best for predicting the prognosis or evaluating the tumor response after chemotherapy or radiotherapy should be investigated using more cases.

In conclusion, Glut1 and pStat3 are associated with the ^18^F-FDG uptake mechanism of NSCLC, and ^18^F-FDG uptake may predict the pStat3 expression level. TLG, MTV, and Glut1 may be independent prognostic factors in resected NSCLC patients.

## MATERIALS AND METHODS

### Cell culture and reagents

Human lung cancer cell lines were maintained under a humidified atmosphere of 5% CO_2_ at 37°C in RPMI 1640 medium (HCC827, EBC1, NCI-H1993, Calu1, A549, NCI-H1975, PC9) supplemented with 10% fetal bovine serum (FBS). EBC1, A549, NCI-H1975, and PC9 were kindly provided by M. Ono (Kyushu University, Fukuoka, Japan). Calu1 was kindly provided by Kazuhiko Nakagawa (Kindai University, Osaka, Japan). HCC827 and NCI-H1993 were purchased from ATCC (Manassas, VA, USA). Cell cultures were routinely confirmed to be free of mycoplasma contamination with the use of a Mycosensor QPCR Assay Kit (Agilent Technologies, Santa Clara, CA, USA). The small interfering RNAs (siRNAs) corresponding to Glut1 and a non-specific siRNA (control) were purchased from Nippon Gene (Tokyo). Cells were transfected with siRNA duplexes using Lipofectamine RNAiMAX and Opti-MEM (Invitrogen, Carlsbad, CA, USA) according to the manufacturer's recommendations.

### Patients

During the period from December 2006 to May 2012, 202 NSCLC patients underwent ^18^F-FDG PET followed by surgical resection. Our inclusion criteria for the patients were as follows: Patients who had undergone (1) complete curative surgical resection and complete mediastinal lymph node resection, with (2) histopathological findings confirming adenocarcinoma or squamous cell carcinoma. The patients who underwent a partial lobectomy (*n*=13) or neoadjuvant chemotherapy before surgery (*n*=9) or who were not followed up at our institution (*n*=9) were excluded. Patients with other histopathological types (*n*=11), those with a past history of malignant tumors (*n*=10), and those with non-useable ^18^F-FDG PET data due to trouble of data (*n*=10) were excluded. Our final study population was 140 NSCLC patients with a median age of 73 (range 42–91) years. The characteristics of the patients are summarized in Table 5. The pathological TNM stages were established using the International System for Staging Lung Cancer adopted by the American Joint Committee on Cancer [31]. This study was conducted according to the Declaration of Helsinki, and was approved by our institutional review board. The need for written informed consent was waived for this retrospective study.

**Table 5:** The characteristic of NSCLC patients

	Number
Gender	
Men	85
Women	55
pStage	
IA	67
IB	24
IIA	19
IIB	6
IIIA	17
IIIB	7
Histological type	
Adeno	114
Sq	26
Smoking Status	
Never Smoker	63
Smoker	77
Chemotherapy after surgery	
Paclitaxel	3
Docetaxel	3
UFT	33
CDDP based chemotherapy	12
CBDCA based chemotherapy	1

### ^18^F-FDG PET imaging acquisition

A dedicated full-ring PET scanner (Allegro, Philips Medical Systems, Cleveland, OH, USA) were used for data acquisition. Before the ^18^F-FDG injection, the patient fasted for 4 hr. The median blood glucose level was 105 mg/dL (range 73–180). The patient was administered a median 282 MBq (range 170–370) of ^18^F-FDG via the antecubital vein. The patient rested quietly for approximately 60 min after the ^18^F-FDG injection. PET emission scans of the areas from the level of the auditory meatus to the mid-thigh were acquired with a time of 2 min 30 sec per cradle position using a three-dimensional acquisition mode. Transmission scans were carried out for all patients to provide attenuation correction with a ^137^Cs point source. After both the transmission and emission images were obtained, the images were reconstructed using the standard normal reconstruction protocol based on a 3D-RAMURA (Philips Eindhoven, The Netherlands) for PET.

### ^18^F-FDG PET image analysis

^18^F-FDG-PET images were displayed on a PET view workstation (Philips Medical Systems) on which a VOI was drawn over the entire abnormal uptake of the primary tumor to include a large amount of radioactivity on axial images semiautomatically. A threshold of 40% of the maximum peak activity within the lesions was used to delineate the MTV [27, 28]. The border of the VOI was adjusted manually by overlap with the adjacent ^18^F-FDG-avid structures, physiological uptake, or lesions. The SUVmax, SUVmean, MTV and TLG of each lesion were calculated and recorded on the workstation.

### Immunohistochemical staining

Histopathological specimens were obtained from surgically resected samples. Immunohistochemistry staining was performed. Paraffin-embedded tissue samples were cut at 4 μm and examined on a coated slide glass, and labeled with the following antibodies using the BenchMark XT (Ventana Automated Systems, Tucson, AZ, USA) and a Bond-III autostainer (Leica Microsystems, Newcastle, UK): Glut1 (1:100, Thermo Scientific, Rockford, IL, USA), pStat1 (1:400, Cell Signaling Technology, Beverly, MA, USA), and pStat3 (1:200, Cell Signaling Technology).

For Glut1, the BenchMark XT was used. Briefly, each slide was heat-treated using Ventana's CC1 retrieval solution for 30 min, and incubated with the Glut1 antibody for 30 min. This automated system used the streptavidin biotin complex method with 3,3' diaminobenzidine (DAB) as the chromogen (Ventana UltraVIEW DAB detection kit). Immunostaining for pStat1 and pStat3 were performed on the same fully automated Bond-III system using onboard heat-induced antigen retrieval with epitope retrieval solution 2 (ER2) for 30 min and a Refine polymer detection system (Leica Microsystems) with horseradish peroxidase (HRP)-polymer as the secondary antibody and DAB.

The immunohistochemical staining examination result for Glut1 was evaluated as positive according to cytoplasm/membrane reactivity, and pStat1 and pStat3 were evaluated as positive according to the nucleus reactivity. We evaluated the intensity of immunostaining as follows: 0, no staining; 1+, weak staining of <10% cancer cells; 2+, moderate staining of 10%–50% cancer cells; 3+, strong staining of >50% cancer cells. All immunohistochemical staining examination results were evaluated by two experienced observers who were blind to the condition of the patients. We calculated the respective scores for Glut1, pStat1, and pStat3 using the intensity multiplied by the immunostained area.

### Statistical analysis

DFS was defined as the period from the date of the patient's surgery until the date of disease recurrence or death. OS was defined as the period between the dates of the surgery and death due to any cause. We classified the patients into pairs of groups: those with low and high values using the optimal cut-off value derived from the 25th and 75th percentiles of the interquartile range (IQR; q1–q3) of the PET parameters and molecular markers expression.

The relationships between all PET parameters and various clinicopathological variables or molecular markers were evaluated using the Wilcoxon rant test and Spearman's rank correlation test. Using the cut-off values, we estimated the survival functions of OS and those of DFS for the low and high groups by the Kaplan-Meier method, and we compared these functions by the log-rank test.

We used a Cox proportional hazard model (continuous PET parameter and molecular marker model) to evaluate the effects of PET parameters and molecular markers while adjusting for potential confounding factors. Differences at p<0.05 were regarded as significant. All analyses were conducted using SAS ver. 9.2 software (SAS Institute, Cary, NC, USA) and R ver. 2.13.0.

## Abbreviations

TNM: tumor-node-metastasis
SUVmax: maximum standardized uptake value
SUVmean: mean standardized uptake value
TLG: total lesion glycolysis
MTV: metabolic tumor volume
Glut1: Glucose transporter-1
Stat1: Signal transducer and activator of transcription-1
pStat1: phosphorylated Stat1
JAK: Janus-activated kinase
INF: interferon
EGFR: epidermal growth factor receptor
pStat3: phosphorylated Stat3
pStage: pathological stage
HR: hazard ratio
CI: confidence interval
HIF-1α: hypoxia-inducible growth factor-1α
VEGF: vascular endothelial growth factor
mTOR: mammalian target of rapamycin
PTEN: phosphatase and tensin homolog
3D-RAMURA: 3D row-action maximum likelihood algorithm
VOI: volumetric region of interest
DFS: Disease-free survival
OS: Overall survival
UFT: tegaful-uracil
CDDP: cisplatin
CBDCA: carboplatin
Adeno: adenocarcinoma
Sq: squamous cell carcinoma
*R*: Spearman rank coefficient
q1-q3: the 25th and 75th percentiles of the interquartile range
MIP: Maximum intensity projection.
